# Irradiation-Induced Structural Evolution and Functional Applications of Carbon-Based Materials: A Review

**DOI:** 10.3390/nano16181143

**Published:** 2026-09-11

**Authors:** Guang Hu, Kuankuan Liu, Jing Tang, Tingting Zhou, Yitong Zhou, Yiheng Guo, Junqi Wang

**Affiliations:** 1 School of Nuclear Science and Technology, Xi’an Jiaotong University, Xi’an 710049, China; guanghu@xjtu.edu.cn; 2 School of Human Settlements and Civil Engineering, Xi’an Jiaotong University, Xi’an 710049, China; lk20010422@stu.xjtu.edu.cn (K.L.); j.tang@stu.xjtu.edu.cn (J.T.); 372498563@stu.xjtu.edu.cn (T.Z.); zhouyitong@stu.xjtu.edu.cn (Y.Z.); g2436390653@stu.xjtu.edu.cn (Y.G.)

**Keywords:** carbon-based materials, irradiation, radiation damage, defect engineering, structural evolution, functional applications

## Abstract

Carbon-based materials exhibit diverse structural responses to irradiation owing to their distinct dimensionality, degree of graphitization, surface chemistry, and pore architecture. Although irradiation has traditionally been regarded as a source of structural damage, increasing evidence demonstrates that controlled irradiation can be deliberately utilized to tailor defects, surfaces, interfaces, and pore structures, thereby enabling desirable functional properties. This review summarizes recent progress in the irradiation-induced structural evolution and functional applications of four representative carbon-based materials, including graphene-based materials, carbon nanotubes, carbon fibers, and activated carbon/biochar. Particular attention is given to the characteristic irradiation responses of different carbon architectures. In graphene, irradiation predominantly induces vacancies, reconstructed defects, and surface functionalization, providing active sites for environmental remediation. Carbon nanotubes additionally undergo inter-tube cross-linking and welding, enabling enhanced mechanical performance and tunable electronic properties. For carbon fibers, irradiation mainly regulates surface chemistry and fiber matrix interactions, facilitating interface engineering in high-performance composites. In activated carbon and biochar, irradiation modifies pore accessibility, structural disorder, and surface functional groups, thereby influencing adsorption and electrochemical performance. These distinct responses demonstrate that irradiation can evolve from a conventional damage process into a controllable materials-engineering strategy when appropriate irradiation conditions are employed. Finally, current challenges associated with optimal irradiation conditions, quantitative defect identification, and irradiation structure–property relationships are discussed. Based on these distinct responses, we propose an architecture-dependent irradiation–structure–function (A-ISF) framework that links the initial carbon architecture and irradiation conditions to dominant energy-deposition mechanisms, structural evolution pathways, property modulation, and ultimately functional applications. Within this framework, irradiation engineering is interpreted as a competition between beneficial structural modification and excessive radiation damage, giving rise to an application-dependent optimal irradiation window.

## 1. Introduction

Carbon-based materials constitute one of the most diverse families of functional and structural materials because carbon atoms can form stable sp, sp^2^, and sp^3^ hybridized configurations, giving rise to structures ranging from highly ordered graphitic lattices to disordered porous carbon frameworks [1,2]. Among them, graphene-based materials, carbon nanotubes, carbon fibers, activated carbon, and biochar have attracted extensive attention owing to their distinctive electrical, mechanical, thermal, and surface properties. Graphene possesses a 2D sp^2^-hybridized lattice with high carrier mobility and accessible surface areas [3], whereas carbon nanotubes combine a one-dimensional tubular architecture with exceptional mechanical and electrical properties [4]. Carbon fibers are particularly attractive for lightweight structural composites because of their high specific strength and stiffness [5], while activated carbon and biochar possess abundant pores and chemically active surfaces suitable for adsorption and electrochemical processes [6]. Consequently, these carbon materials have been extensively investigated in environmental remediation, energy storage, electronics, sensing, and advanced structural composites [7].

Despite these attractive properties, the performance of carbon materials is strongly governed by their defect structure, surface chemistry, pore architecture, and interfacial characteristics. Conventional modification strategies therefore commonly employ chemical oxidation, heteroatom doping, thermal treatment, plasma modification, or surface grafting to tailor these features [8]. However, chemical modification may involve strong acids or oxidizing agents and can introduce impurities, whereas high-temperature treatment is energy intensive and may cause undesired structural changes. Irradiation provides an alternative approach because energetic photons or particles can transfer energy directly to carbon atoms or surrounding molecules, thereby inducing bond cleavage, atomic displacement, radical formation, and subsequent structural reconstruction without necessarily introducing additional chemical reagents. Depending on the radiation source and treatment conditions, γ-rays, electron beams, and energetic ions can therefore be used to regulate carbon structures over different length scales [9,10].

Historically, irradiation of carbon materials has primarily been investigated from the perspective of radiation damage, particularly because carbon-based components and devices may be exposed to energetic particles and ionizing radiation in nuclear reactors, spacecraft, accelerators, and other radiation environments [11]. Energetic irradiation can displace carbon atoms from their equilibrium lattice positions and generate vacancies, interstitials, reconstructed defects, and eventually amorphous regions, potentially degrading the electrical and mechanical properties of carbon materials [12]. For example, γ irradiation has been shown to produce distinctly different structural responses in graphite and multi-walled carbon nanotubes, illustrating that radiation effects depend strongly on the initial carbon architecture [13]. More generally, the irradiation response is affected by radiation type, energy, dose, temperature, surrounding atmosphere, and intrinsic material structure, making irradiation damage a highly material-dependent process.

Increasing evidence, however, demonstrates that irradiation-induced structural changes are not necessarily detrimental. When irradiation parameters are appropriately controlled, defects that were traditionally regarded as radiation damage can instead act as deliberately introduced functional sites [14]. At the atomic scale, irradiation can generate vacancies and reconstructed carbon rings; at larger length scales, these defects may migrate and coalesce to form nanopores or trigger substantial structural transformations. Modern atomic-resolution electron microscopy has directly revealed processes including carbon-atom displacement, divacancy reconstruction, adatom migration, and irradiation-induced transformation of carbon nanotube structures [9]. In addition, irradiation-generated radicals can alter surface functional groups, promote grafting reactions, or strengthen interfaces between adjacent phases. Thus, the conceptual role of irradiation is gradually shifting from passive radiation damage toward active radiation engineering, in which controlled irradiation is intentionally employed to manipulate defects, surfaces, pores, and interfaces. Importantly, this transition cannot be described by a single universal irradiation mechanism because different carbon architectures exhibit fundamentally different responses. In graphene, irradiation predominantly generates vacancies, reconstructed defects, and surface chemical changes within the 2D lattice [10]. In carbon nanotubes, the curved tubular geometry enables additional phenomena such as inter-tube cross-linking, welding, and irradiation-induced shape transformation [9]. For carbon fibers, irradiation effects are particularly important at the fiber surface and fiber-matrix interface, whereas in activated carbon and biochar, their intrinsically disordered and porous structures make pore accessibility and surface functional groups particularly sensitive to irradiation. These structural differences ultimately determine whether irradiation influences adsorption, mechanical load transfer, electrical transport, interfacial adhesion, or electrochemical behavior [15,16]. Previous reviews have discussed γ-ray-induced transformations of carbon systems or electron/ion irradiation effects in graphene and carbon nanotubes, confirming the highly structure-dependent nature of radiation responses. However, existing reviews have largely concentrated either on particular irradiation sources or on selected nanocarbon systems, while the connection between irradiation-induced structural evolution and the subsequent functional utilization of different classes of carbon materials has received comparatively less systematic attention.

Accordingly, this review provides a concise overview of irradiation-induced structural evolution and functional applications in four representative classes of carbon-based materials: graphene-based materials, carbon nanotubes, carbon fibers, and activated carbon/biochar. Particular attention is given to how their different dimensionalities and structural characteristics determine their responses to irradiation and how these structural changes can subsequently be exploited for functional applications. Rather than considering irradiation exclusively as a source of material degradation, this review emphasizes the transition from radiation-induced damage to controllable structural modification, including defect engineering in graphene, inter-tube cross-linking in carbon nanotubes, interface regulation in carbon fibers, and pore/surface-chemistry engineering in activated carbon and biochar. Finally, the remaining challenges associated with irradiation-condition optimization, defect identification, and irradiation structure–property relationships are discussed to provide perspectives for the rational design of irradiation-modified carbon materials.

## 2. Irradiation Effects and Applications of Carbon-Based Materials

Carbon-based materials, including graphene, carbon nanotubes, carbon fibers, activated carbon (AC) and biochar, have attracted tremendous attention owing to their outstanding physicochemical properties, including excellent electrical conductivity, high specific surface area, superior mechanical strength, and remarkable chemical stability [17]. These unique properties originate from their diverse carbon hybridization states (sp^2^ and sp^3^) and highly tunable microstructures. Depending on their dimensionality and crystallinity, carbon materials exhibit significantly different responses to external irradiation; however, their structural evolution is fundamentally governed by the interaction between energetic particles and carbon atoms [18]. Unlike conventional thermal or chemical modification methods, irradiation provides a non-contact and highly controllable strategy for tailoring the atomic structure of carbon materials. High-energy γ-rays, electron beams, ion beams, and neutrons deposit energy into carbon lattices through elastic collisions and inelastic electronic interactions [19]. Depending on the radiation source and irradiation conditions, this energy deposition may induce atomic displacement, electronic excitation, ionization, bond breaking, and defect reconstruction, thereby modifying both the crystal structure and surface chemistry of carbon materials [9,10]. As a consequence, irradiation has gradually evolved from a technique traditionally associated with radiation damage into an effective defect-engineering strategy for regulating the physicochemical properties of carbon-based materials.

The primary irradiation-induced process is the formation of atomic-scale defects. When the transferred kinetic energy exceeds the displacement threshold energy of carbon atoms, atoms can be displaced from their lattice sites, producing vacancies, interstitial atoms, and Frenkel pairs. These primary defects rarely remain isolated. Instead, they continuously migrate, recombine, and aggregate under sustained irradiation, resulting in more complex defect configurations such as divacancies, Stone-Wales defects, nanopores, reconstructed edges, and locally amorphous regions [20]. Owing to the exceptional flexibility of graphitic networks, many of these defects undergo spontaneous structural reconstruction, leading to the formation of energetically favorable configurations rather than complete lattice collapse. This self-reconstruction capability distinguishes carbon materials from many metallic systems and constitutes the theoretical basis for irradiation-assisted defect engineering. For low-linear-energy-transfer (LET) radiation such as γ-rays and high-energy electrons, ionization and electronic excitation dominate the interaction process. Instead of directly displacing large numbers of carbon atoms, irradiation generates abundant secondary electrons and reactive radicals, which subsequently induce bond cleavage and rehybridization reactions. In oxidative environments, these reactions frequently promote the formation of oxygen-containing functional groups, including hydroxyl (-OH), carbonyl (C=O), epoxy (C-O-C), and carboxyl (-COOH) groups [21,22]. The incorporation of these surface functionalities significantly enhances surface polarity, wettability, adsorption affinity, and interfacial chemical reactivity, thereby improving the catalytic and adsorption performance of carbon materials without introducing severe structural degradation [23,24].

Although radiation-induced defect generation follows a common physical principle, the structural evolution and functional response of different carbon-based materials vary considerably because of their distinct dimensionality, crystallinity, and bonding characteristics [25]. Therefore, representative carbon-based materials are discussed individually in the following sections to highlight their unique irradiation behaviors, defect evolution mechanisms, and corresponding structure–property relationships. Furthermore, γ-rays, electron beams, and ion beams can all cause structural changes in carbon materials, but their energy deposition mechanisms and the resulting structural responses are significantly different. Therefore, in Table 1, we have provided a comparative overview of the typical irradiation mechanisms and structural effects of these irradiation sources.

**Figure 1:**
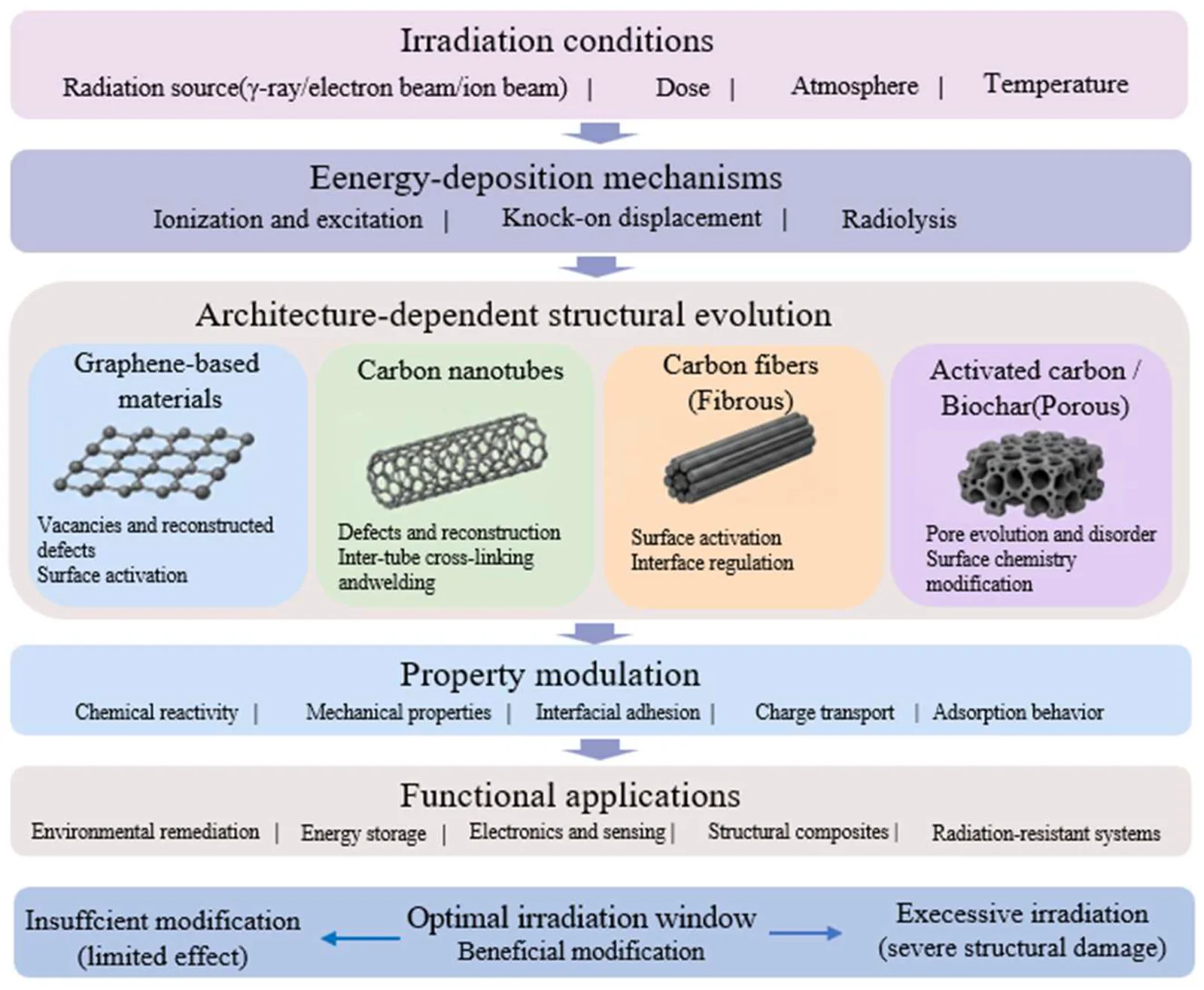
Architecture-dependent irradiation–structure–function (A-ISF) framework for carbon-based materials, illustrating the relationships among irradiation conditions, structural evolution, property modulation, and functional applications.

Based on the studies reviewed here, an architecture-dependent irradiation–structure–function (A-ISF) framework is proposed to link irradiation conditions, structural evolution, property modulation, and functional applications (Figure 1). Irradiation type, energy, dose, dose rate, atmosphere, and temperature determine the energy-deposition processes, while the dimensionality, crystallinity, surface characteristics, and pore structure of carbon materials govern their subsequent structural responses [9,10,18,19].

### 2.1. Graphene-Based Materials

Graphene is a two-dimensional (2D) carbon nanomaterial consisting of a single atomic layer of carbon atoms arranged in a hexagonal honeycomb lattice through sp^2^ hybridization. Since its first isolation in 2004 [26], graphene has attracted tremendous interest because of its exceptional carrier mobility, superior thermal conductivity, excellent mechanical strength, and extremely high theoretical specific surface area. These unique characteristics originate from its highly conjugated π-electron system and atomically thin structure, making graphene one of the most representative carbon-based materials for irradiation modification [27]. Unlike bulk graphite, every carbon atom in graphene is directly exposed to incident radiation, enabling efficient energy transfer and making graphene particularly sensitive to atomic-scale structural evolution induced by energetic particles. Consequently, even relatively low irradiation doses can generate detectable changes in lattice structure, electronic configuration, and surface chemistry, providing an ideal platform for radiation-induced defect engineering. Radiation-induced structural evolution in graphene generally begins with the formation of point defects. Depending on the radiation source and deposited energy, energetic particles transfer sufficient kinetic energy to carbon atoms to overcome the displacement threshold, resulting in the generation of vacancies, divacancies, and other lattice defects [28]. Compared with traditional chemical oxidation or plasma treatment, irradiation offers a non-contact and highly controllable approach that can retain the inherent two-dimensional structure of graphene to a certain extent while introducing defects. Therefore, irradiation has become an effective approach for tailoring the defect density, electronic structure, and chemical activity of graphene without introducing significant contamination [10,29].

#### 2.1.1. Radiation-Induced Structural Evolution

The formation of atomic-scale defects represents the earliest stage of graphene structural evolution under irradiation. Regardless of the irradiation source, energetic particles transfer energy to the graphene lattice through nuclear collisions and/or electronic excitation. Once the transferred energy exceeds the displacement threshold of carbon atoms, lattice atoms are displaced from their equilibrium positions, leading to the formation of vacancies, divacancies, and other point defects. These defects subsequently undergo migration and reconstruction, eventually evolving into more stable configurations such as Stone-Wales defects, reconstructed divacancies, and nanopores [14]. Besides lattice reconstruction, irradiation also profoundly alters the surface chemistry of graphene. The vacancies and dangling bonds generated during irradiation possess significantly higher chemical activity than the intact sp^2^ carbon lattice, making them preferential sites for the adsorption of oxygen-containing species when irradiation is performed in air or aqueous environments. Consequently, irradiation simultaneously induces structural defects and chemical functionalization, which together dominate the physicochemical evolution of graphene. Rather than being independent processes, defect formation and surface functionalization occur synergistically throughout irradiation, ultimately determining the electronic structure and chemical reactivity of graphene [28].

**Figure 2:**
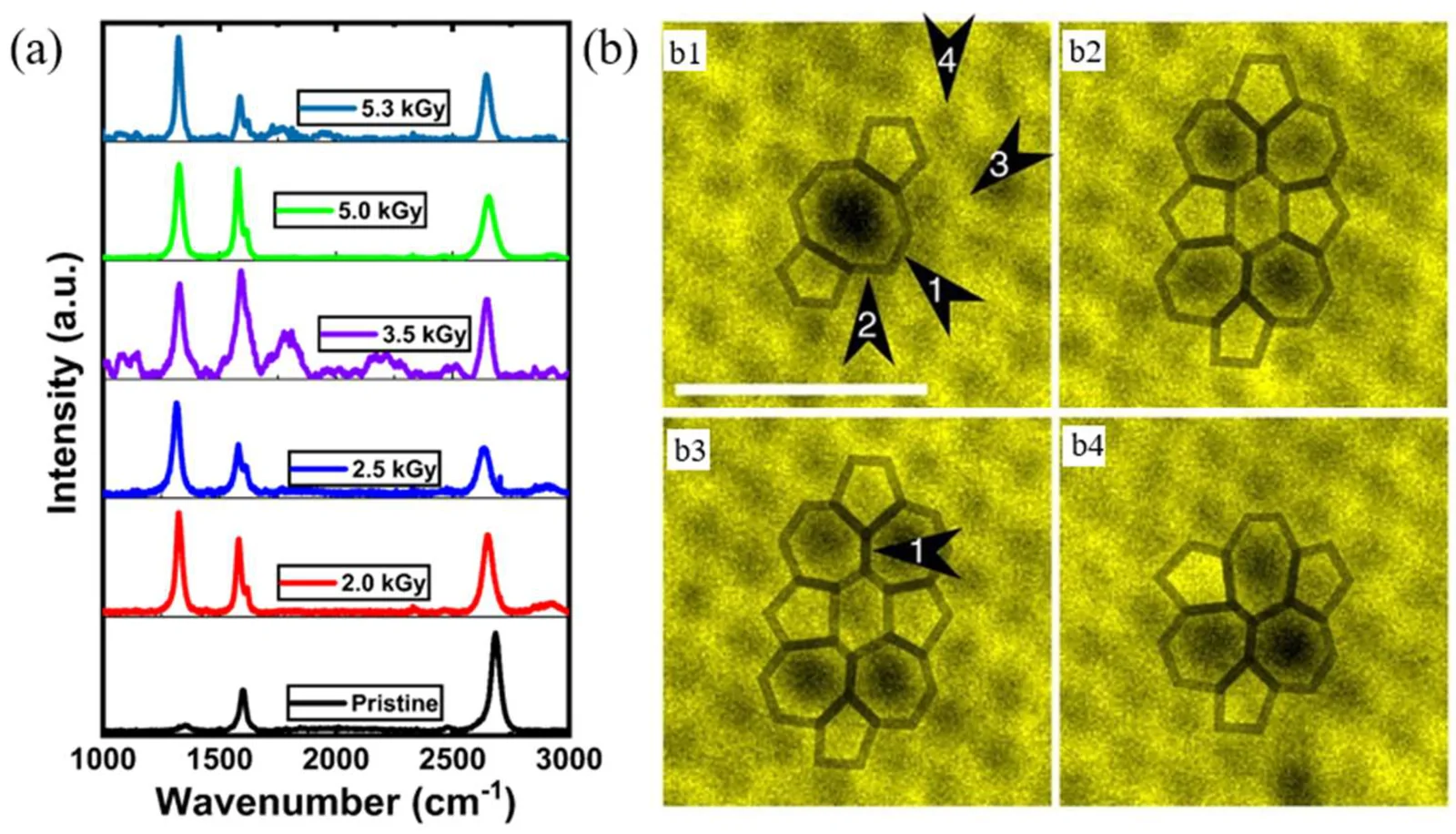
Irradiation-induced structural evolution of graphene. (**a**) Raman spectra of γ-irradiated monolayer CVD graphene at different irradiation doses (Adapted from Chavda et al. [27]). (**b**) Consecutive atomic-resolution STEM images showing electron-beam-driven transformations of a divacancy in graphene at 60 kV, with overlaid bond structures highlighting the reconstructed configurations. Scale bars: 1 nm. (Adapted from Kotakoski et al. [32]).

Representative evidence was provided by Chavda et al. [27], who systematically investigated the structural evolution of monolayer CVD-grown graphene subjected to ^60^Co γ ray irradiation. Raman spectroscopy (Figure 2a) demonstrated that the D band, which was nearly absent in pristine graphene, gradually emerged and intensified with increasing irradiation dose, indicating the progressive generation of irradiation-induced point defects. Meanwhile, the evolution of the *I*_D_/*I*_G_ ratio suggested a transition from graphene with a highly ordered sp^2^ carbon lattice to a nanocrystalline defective structure at low irradiation doses, followed by partial amorphization at higher doses due to excessive defect accumulation [30]. Complementary XPS analysis further revealed a gradual decrease in graphitic C-C bonds accompanied by a continuous increase in oxygen-containing functional groups, including C-O, C-OH and -COOH, demonstrating that irradiation-generated vacancies serve as preferential adsorption sites for oxygen species. Density functional theory (DFT) calculations further confirmed that local lattice distortion surrounding vacancy defects reduced the adsorption energy of oxygen-containing species, thereby facilitating surface functionalization [31]. These results collectively indicate that γ-ray irradiation can simultaneously regulate lattice defects and surface chemistry, while maintaining a portion of the inherent framework structure of two-dimensional graphene. Therefore, it is an effective controllable defect engineering strategy [10].

In contrast to γ-ray irradiation, electron irradiation can drive the structural evolution of existing defects in graphene through bond rotation. For instance, Kotakoski et al. [32] directly observed the reconfiguration and migration process of a double vacancy in a monolayer graphene using a 60 kV atomic-resolution scanning transmission electron microscope (STEM) (Figure 2b). The continuous images showed a transition between the 5–8–5, 5555–6–7777, and 555–777 double vacancy configurations after the reconfiguration. These bond rotations driven by the electron beam changed the configuration and position of the defects, causing their migration within the graphene lattice. These observations provide direct atomic-scale evidence for defect reconfiguration under electron irradiation, complementing the spectral characterization of irradiation-induced structural changes.

#### 2.1.2. Representative Applications of Irradiation-Modified Graphene

**Figure 3:**
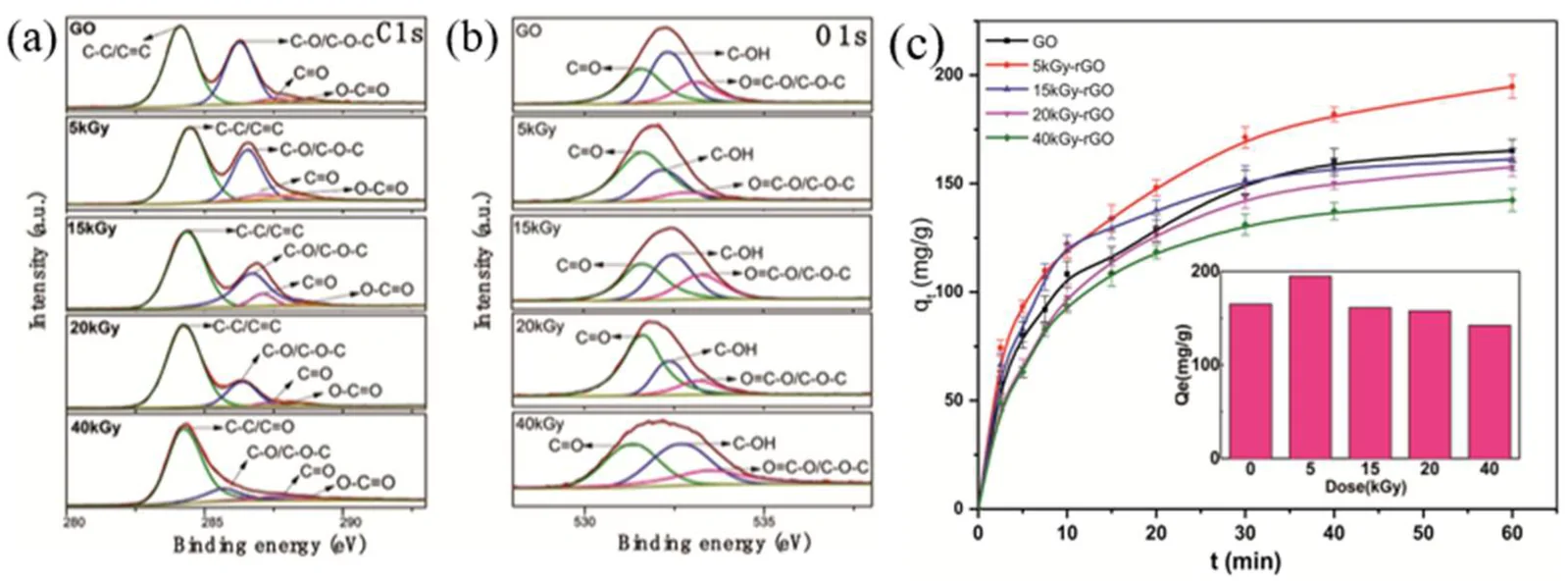
Electron-beam-irradiation-induced regulation of graphene oxide surface chemistry and Pb(II) adsorption performance. (**a**,**b**) High-resolution C 1s and O 1s XPS spectra of GO/rGO subjected to different electron-beam irradiation doses. (**c**) Pb(II) adsorption kinetics of GO/rGO at different irradiation doses; the inset shows the corresponding equilibrium adsorption capacities. (Adapted from Yang et al. [35]).

The irradiation-induced structural evolution discussed in the previous section has substantially expanded the practical applications of graphene-based materials [33]. Among the various application scenarios that have been reported, environmental remediation has become the most intensively studied field, as irradiation can simultaneously increase the defect density and the surface oxygen-containing functional groups. Moreover, depending on the irradiation conditions and the surrounding environment, the inherent framework structure of graphene can be partially maintained. These structural modifications significantly improve the interaction between graphene and dissolved contaminants, making irradiation-modified graphene a promising adsorbent for heavy metals and radionuclides. Representative studies are summarized below [34]. Yang et al. [35] reported a typical example in which they used electron beam irradiation to controllably adjust the oxygen content and surface chemical properties of graphene oxide. By varying the irradiation dose from 5 to 40 kGy, they obtained reduced graphene oxide (rGO) with systematically different C/O ratios and oxygen-containing functional groups (Figure 3a). They also found that the surface chemical regulation induced by irradiation significantly affected the subsequent adsorption ability of lead (II) from aqueous solutions. In the irradiated samples, rGO treated with 5 kGy irradiation exhibited the most favorable adsorption behavior, with the maximum Pb(II) adsorption capacity reaching 194.76 mg g^−1^ (Figure 3b). These results indicate that electron beam irradiation can not only be used to modify the chemical structure of graphite-based materials, but also to regulate their adsorption performance by controlling the abundance and distribution of surface oxygen-containing functional groups.

Besides heavy-metal removal, irradiation-modified graphene has also demonstrated considerable potential for radionuclide remediation. Because irradiation generates abundant oxygen-containing functional groups and defect sites capable of forming stable surface complexes with actinides and fission products, GO has become an attractive adsorbent for radioactive wastewater treatment. Recent studies have shown that U(VI), Eu(III), Am(III), and other radionuclides can be efficiently immobilized through coordination with -COOH and -OH groups located at graphene defect sites [36]. Surface complexation modeling combined with spectroscopic analyses further confirmed that the adsorption process is dominated by inner-sphere complexation rather than simple electrostatic attraction, highlighting the importance of irradiation-induced surface functionalization for radionuclide capture.

**Figure 4:**
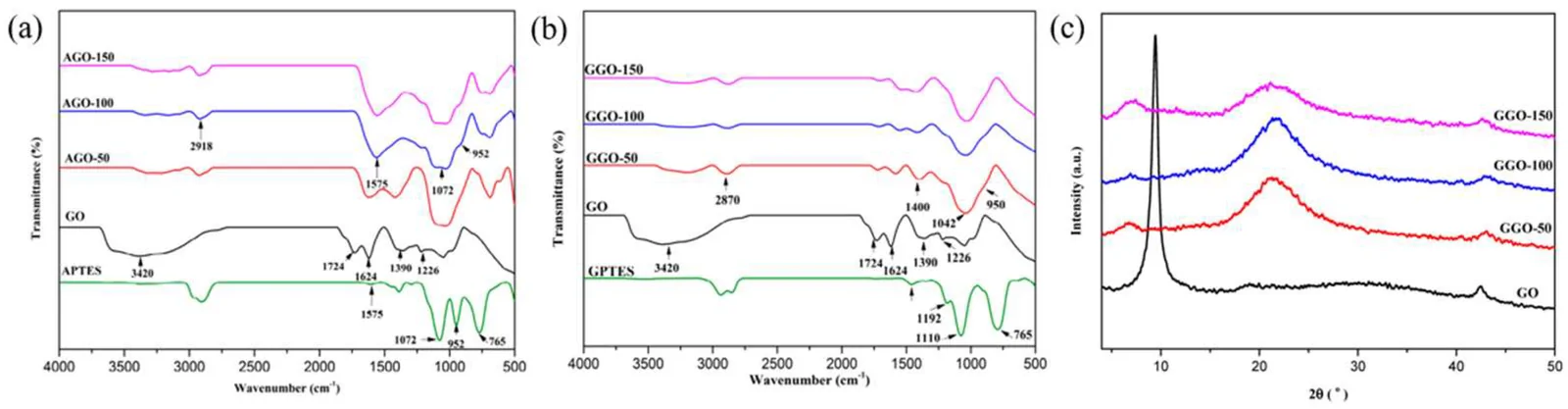
(**a**) FTIR spectra of GO and 3-aminopropyltriethoxysilne functionalized-GO (AGO-50, AGO-100 and AGO-150); (**b**) FTIR spectra of GO and 3-glycidyloxypropyltrimethoxysilane functionalized-GO (GGO-50, GGO-100 and GGO-150); (**c**) XRD spectra of GO and 3-glycidyloxypropyltrimethoxysilane functionalized-GO (GGO-50, GGO-100, and GGO-150). (Adapted from Aujara et al. [37]).

More recently, irradiation-modified graphene has attracted increasing attention for the treatment of complex wastewater containing multiple coexisting contaminants, where the simultaneous removal of heavy metals, radionuclides, dyes, and other organic pollutants is often required. Compared with conventional chemically oxidized graphene, irradiation-modified graphene possesses a more controllable defect distribution and surface chemistry, enabling stronger interactions with different classes of contaminants through electrostatic attraction, surface complexation, hydrogen bonding, and π-π interactions [36]. Beyond direct contaminant adsorption, irradiation can also serve as a platform for secondary surface functionalization, thereby broadening the chemical tunability of graphene-based materials. A representative example was reported by Aujara et al. [37], who employed γ-rays to graft organosilane molecules, including 3-aminopropyltriethoxysilane (APTES) and 3-glycidyloxypropyltrimethoxysilane (GPTES), onto the surface of GO. Spectroscopic and structural characterization confirmed successful chemical interactions between the organosilanes and the GO surface, while Raman analysis revealed changes in the defect structure associated with surface functionalization (Figure 4). Importantly, the irradiation-assisted process provides a non-contact route for modifying the physicochemical properties of GO without relying on conventional harsh reduction conditions. This study demonstrates that γ irradiation can serve not only as a source of structural defects but also as an effective platform for radiation-induced surface grafting, thereby expanding the chemical tunability of graphene-based materials for subsequent environmental and interfacial applications.

In addition to laboratory-scale studies, the environmental applicability of graphene-based adsorbents has also been evaluated using real industrial wastewater. Recent investigations have demonstrated that GO can effectively remove multiple heavy-metal ions, including Fe, Cu, Cr, and Pb, from mining wastewater under optimized adsorption conditions, confirming its potential for practical environmental remediation [38]. These studies indicate that irradiation-induced defect engineering, together with appropriate surface functionalization, provides a promising pathway for developing graphene-based adsorbents capable of treating complex wastewater systems containing multiple coexisting contaminants.

### 2.2. Carbon Nanotubes

#### 2.2.1. Radiation-Induced Structural Evolution

Carbon nanotubes are one-dimensional carbon nanomaterials consisting of seamlessly rolled graphene sheets with nanometer-scale diameters and exceptionally high aspect ratios. Depending on the number of concentric graphene cylinders, carbon nanotubes can be classified into single-walled carbon nanotubes and multi-walled carbon nanotubes [4,39]. Owing to their unique tubular geometry, outstanding mechanical strength, excellent electrical conductivity, and high chemical stability, carbon nanotubes have been extensively investigated for applications in structural composites, nanoelectronics, sensors, and energy-storage devices. Unlike graphene, whose irradiation response is mainly dominated by defect formation on a 2D basal plane, carbon nanotubes exhibit a much richer structural evolution under irradiation because their curved tubular walls and multi-shell configurations facilitate not only defect generation but also tube-tube cross-linking, welding, and structural reconstruction [9]. Consequently, irradiation has become an effective strategy for tailoring both the intrinsic structure of individual carbon nanotubes and the interfacial interactions within carbon nanotube assemblies.

Under irradiation, the earliest structural response of carbon nanotubes is the formation of vacancy-related defects through knock-on displacement of carbon atoms. Similar to graphene, energetic electrons or ions transfer sufficient kinetic energy to carbon atoms to create single vacancies and divacancies. However, because of the intrinsic curvature of carbon nanotube walls, these irradiation-induced defects are energetically less stable and readily undergo atomic reconstruction [40]. HRTEM observations have demonstrated that vacancies continuously migrate along the nanotube wall and transform into reconstructed defects containing non-hexagonal carbon rings, thereby partially restoring the structural stability of the nanotube [41,42]. The defect evolution strongly depends on irradiation energy, tube diameter, wall number, and irradiation temperature, indicating that irradiation parameters can be used to precisely regulate the defect density and structural evolution of carbon nanotubes [43,44]. More importantly, irradiation induces structural phenomena that are unique to carbon nanotubes. Owing to the close contact between adjacent nanotubes within bundles or fibers, irradiation-generated vacancies and dangling bonds can react with neighboring nanotubes to form inter-tube covalent bonds, resulting in cross-linking and welding between adjacent carbon nanotubes [45]. Compared with simple defect formation, this process significantly enhances load transfer between nanotubes and effectively suppresses interfacial sliding, thereby improving the mechanical integrity of carbon nanotube assemblies. Both theoretical simulations and experimental observations have confirmed that appropriate electron or ion irradiation promotes the formation of covalent bridges between neighboring carbon nanotubes, whereas excessive irradiation introduces severe structural damage and eventually leads to tube collapse or amorphization [45,46]. Therefore, achieving an optimal balance between defect generation and cross-linking is considered the key to irradiation engineering of carbon nanotube-based materials.

In addition to defect reconstruction and inter-tube cross-linking, irradiation also alters the surface chemistry of carbon nanotubes. The dangling bonds generated around irradiation-induced defects readily react with oxygen- or nitrogen-containing species in the surrounding atmosphere, producing -OH, C=O, and -COOH groups on the nanotube surface [47]. These oxygen-containing functional groups not only improve the dispersion of carbon nanotubes in aqueous media but also provide abundant anchoring sites for polymers, nanoparticles, and metal ions, thereby facilitating subsequent functionalization and composite fabrication [48]. Consequently, irradiation simultaneously regulates the structural integrity, interfacial bonding, and surface chemistry of carbon nanotubes, establishing the structural basis for their diverse functional applications.

#### 2.2.2. Applications of Irradiation-Modified Carbon Nanotubes

The unique irradiation-induced structural evolution of carbon nanotubes, particularly the formation of inter-tube covalent cross-links and reconstructed defects, has significantly expanded their practical applications. Compared with graphene, the most distinctive advantage of irradiation-modified carbon nanotubes lies in the ability to improve load transfer between adjacent nanotubes through irradiation-induced cross-linking. Consequently, current studies have mainly focused on mechanical reinforcement of carbon nanotube fibers and composites, followed by functional modification for electrochemical and catalytic applications. One of the earliest and most representative applications of irradiation-modified carbon nanotubes is the enhancement of mechanical properties through irradiation-induced cross-linking [45]. Although individual carbon nanotubes possess exceptional tensile strength and Young’s modulus, these outstanding intrinsic properties are difficult to translate into macroscopic fibers because adjacent nanotubes interact primarily through weak van der Waals forces. Electron and ion irradiation provide an effective solution by generating vacancies and dangling bonds that subsequently form covalent bridges between neighboring nanotubes. These inter-tube cross-links markedly suppress nanotube slippage and improve stress transfer efficiency, thereby enhancing the mechanical integrity of carbon nanotube fibers and yarns [46].

**Figure 5:**
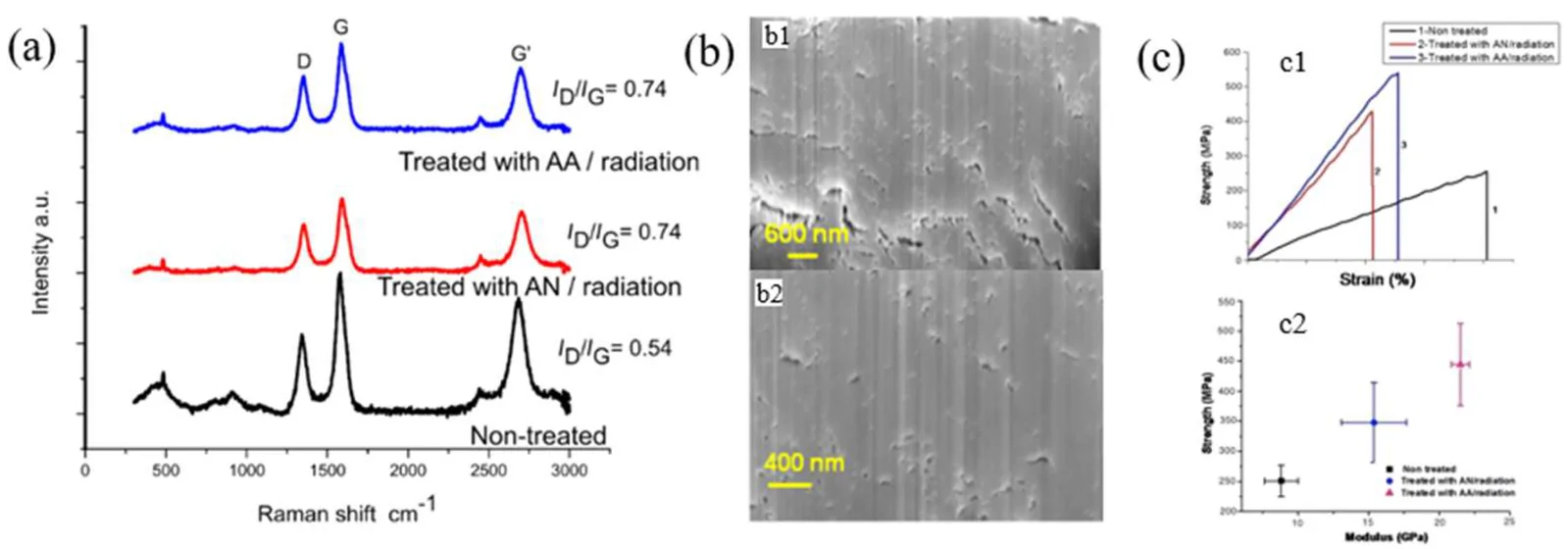
Electron-beam-irradiation-assisted functionalization and mechanical reinforcement of CNT yarns. (**a**) Raman spectra of untreated CNT yarn and CNT yarns treated with AN or AA followed by electron-beam irradiation. (**b**) FIB images showing the internal morphology of AA-treated and irradiated CNT yarn. (**c**) Representative stress–strain curves and tensile strength–modulus relationships of untreated and irradiation-modified CNT yarns. (Adapted from Evora et al. [49]).

The representative study by Evora et al. [49] demonstrated that, in the presence of acrylic acid (AA) or acrylonitrile (AN), a one-step electron beam strategy can be employed to simultaneously functionalize and crosslink carbon nanotube (CNT) yarns. Raman spectroscopy revealed that the ID/IG ratio in untreated CNT yarns was 0.54, which increased to 0.74 after irradiation-assisted modification, indicating that the functionalization process led to an increase in the disorder degree of the carbon nanotube structure (Figure 5a). The field emission ion beam (FIB) observation further showed that after AA-assisted irradiation treatment, the internal structure of the yarns underwent significant modification, and the inter-tube void space decreased (Figure 5b). These structural changes significantly enhanced the interaction between CNTs and inhibited intratube sliding, thereby improving the load transfer performance within the yarn. Therefore, the tensile strength of untreated CNT yarns increased from 251.1 ± 26.5 MPa to 444.5 ± 68.5 MPa, and the modulus rose from 8.79 ± 1.19 GPa to 21.5 ± 0.65 GPa (Figure 5c). These results indicate that irradiation-assisted functionalization and crosslinking provide an effective way to transform the excellent inherent mechanical properties of carbon nanotubes into improved macroscopic yarn performance.

In recent years, irradiation-modified carbon nanotubes have attracted considerable attention for radiation-hardened electronics and sensing devices, owing to their intrinsically robust sp^2^ carbon framework and excellent tolerance toward high-energy radiation. Unlike conventional silicon-based semiconductors, which generally suffer from significant degradation under ionizing radiation, carbon nanotube-based electronic devices can maintain stable electrical performance after high-dose irradiation because irradiation-induced defects are primarily localized within the nanotube network without causing catastrophic structural failure. Controlled γ-ray or EB irradiation can further regulate the carbon nanotube/dielectric interface, suppress charge trapping, and optimize carrier transport, thereby improving both electrical stability and radiation tolerance [50,51].

**Figure 6:**
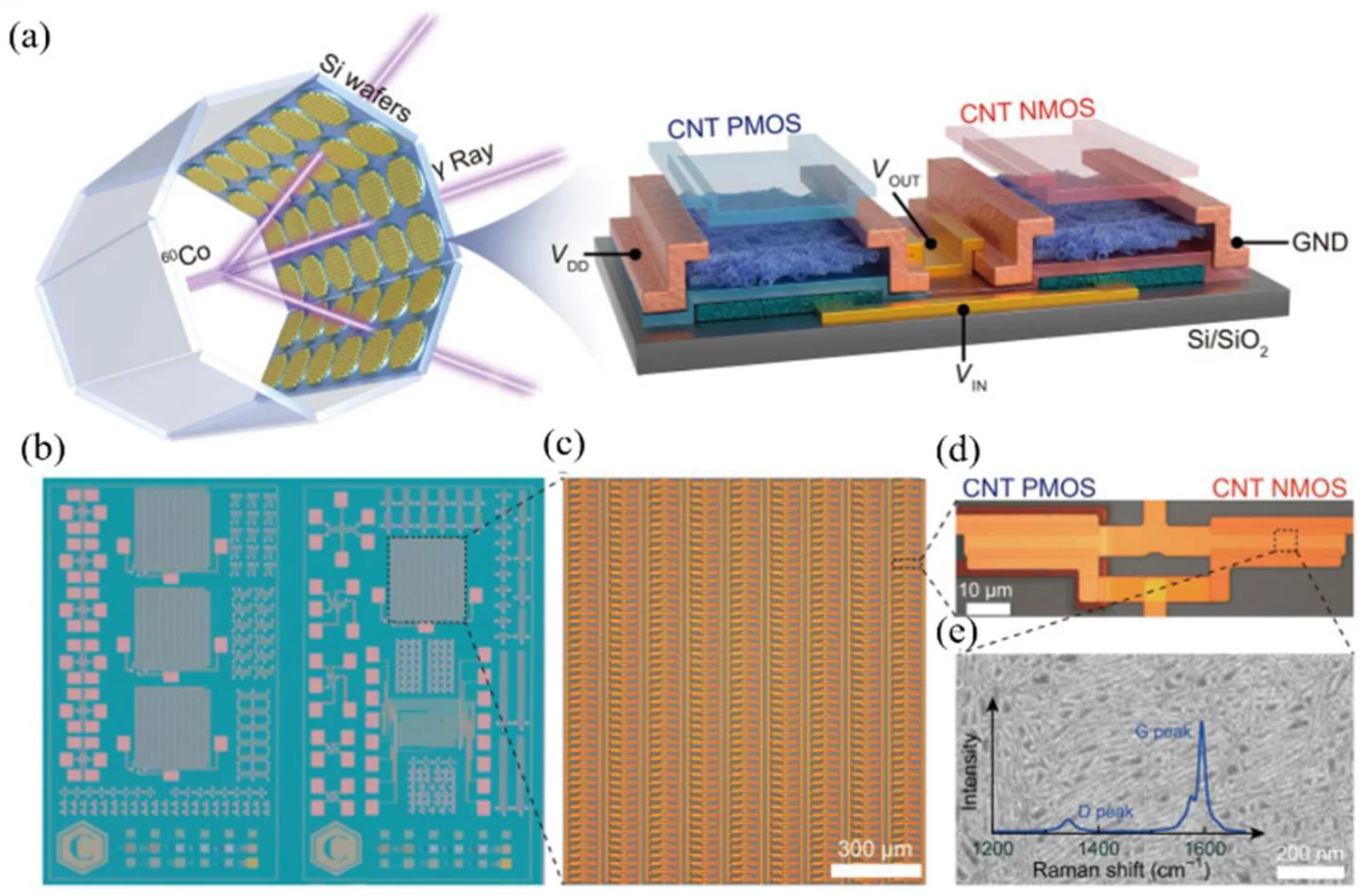
Structure and characterization of large-scale radiation-tolerant CNT CMOS. (**a**) Schematic illustration of γ-ray irradiation and the complementary CNT CMOS architecture. (**b**) Photograph of the fabricated CNT IC devices. (**c**) Optical micrograph of a representative CNT IC. (**d**) Optical micrograph of a CNT CMOS FET. (**e**) SEM image and Raman spectrum of the CNT network film. (Adapted from Zhang et al. [50]).

A representative example was reported by Zhu et al. [51], who systematically investigated the radiation response of top-gated carbon nanotube field-effect transistors under γ-ray irradiation. By independently evaluating the radiation responses of the carbon nanotube channel, gate dielectric, and substrate, the authors demonstrated that the semiconducting carbon nanotube network itself exhibited remarkable resistance to radiation damage, whereas the substrate contributed most of the degradation observed in device performance. More importantly, the carbon nanotube film partially shielded the underlying substrate from irradiation-induced damage, enabling the fabricated carbon nanotube field-effect transistors to withstand total ionizing doses of at least 155 kGy while maintaining stable transistor characteristics. These findings highlighted the intrinsic radiation hardness of carbon nanotube channels and demonstrated their potential for next-generation radiation-resistant electronic devices. More recently, Zhang et al. [50] further extended this concept by fabricating large-scale complementary carbon nanotube integrated circuits (CNT CMOS) for harsh radiation environments (Figure 6). Remarkably, after exposure to 60 kGy(Si) of γ irradiation, various logic gates and ring oscillators still maintained normal rail-to-rail output characteristics with only minimal delay variation. This work demonstrated, for the first time, that carbon nanotube electronics could retain stable operation not only at the individual transistor level but also within large-scale integrated circuits containing more than one thousand carbon nanotube field-effect transistors, representing a major step toward practical radiation-tolerant electronics for aerospace and nuclear applications.

In addition to radiation-hard electronics, irradiation-modified carbon nanotubes have also shown considerable promise for sensing applications. Radiation-induced defect engineering can regulate the conductivity and carrier transport behavior of carbon nanotube networks while simultaneously introducing chemically active sites for molecular adsorption [52]. Benefiting from these characteristics, carbon nanotube -based sensors have been successfully developed for radiation detection and environmental monitoring [53]. For example, Molinari et al. [54] fabricated a conductive electrospun composite containing multi-walled carbon nanotubes and fullerene (C_60_), which exhibited a permanent conductivity increase after EB irradiation and could directly function as an electron-radiation microsensor. The authors demonstrated that the device responded sensitively to irradiation doses as low as 0.02 pC μm^−2^, indicating that carbon nanotube-based conductive networks can be employed not only as radiation-resistant electronic materials but also as active radiation-sensing components.

Overall, these representative studies demonstrate that irradiation enables carbon nanotubes to evolve from conventional conductive nanomaterials into multifunctional electronic building blocks capable of operating under extreme radiation environments [55]. Through controlled defect engineering and interface regulation, irradiation not only improves the radiation tolerance of carbon nanotube-based transistors and integrated circuits but also expands their applications in radiation sensing and intelligent electronic systems, highlighting the unique advantages of carbon nanotubes over conventional semiconductor materials [44].

### 2.3. Carbon Fibers

#### 2.3.1. Radiation-Induced Structural Evolution

Carbon fibers are among the most widely used structural carbon materials owing to their outstanding specific strength, high stiffness, excellent fatigue resistance, and superior thermal stability. They have been extensively employed in aerospace, nuclear engineering, wind energy, and high-performance transportation, where lightweight structures with exceptional mechanical reliability are required [56]. Unlike graphene and carbon nanotubes, whose irradiation responses are primarily governed by defect generation within their graphitic frameworks, the performance of carbon fibers is largely determined by the fiber–matrix interface [57]. Consequently, irradiation modification of carbon fibers mainly focuses on tailoring the surface morphology and interfacial chemistry rather than altering the bulk graphitic structure of the fibers themselves [58].

Exposure to γ-rays, electron beams, or ion beams introduces active radicals and dangling bonds on the carbon fiber surface, leading to localized surface etching and the formation of oxygen-containing functional groups. These surface reactions increase the roughness and surface energy of carbon fibers while simultaneously improving their wettability toward polymer matrices. Spectroscopic characterization using Raman spectroscopy and X-ray photoelectron spectroscopy (XPS) has consistently demonstrated that irradiation promotes the formation of -OH, C=O, and -COOH groups on the fiber surface, whereas scanning electron microscopy (SEM) and atomic force microscopy (AFM) reveal a rougher surface morphology after irradiation. These structural and chemical modifications provide additional anchoring sites for resin infiltration and chemical bonding, thereby establishing the structural basis for enhanced interfacial adhesion between carbon fibers and polymer matrices [59,60]. A representative study by Ma et al. [59] investigated the influence of ^60^Co γ-ray irradiation on polyacrylonitrile (PAN)-based carbon fibers. Raman spectroscopy indicated that irradiation slightly increased the defect density on the fiber surface, while XPS analysis revealed a significant increase in oxygen-containing functional groups. Correspondingly, SEM observations showed that the fiber surface became noticeably rougher without introducing severe structural damage. Mechanical testing further demonstrated that moderate irradiation substantially improved the interlaminar shear strength (ILSS) of carbon fiber/C-O-C composites, whereas excessive irradiation caused degradation of fiber strength because of over-etching and excessive surface damage. These results indicate that an appropriate irradiation dose is essential for balancing surface activation and structural integrity.

#### 2.3.2. Representative Applications of Irradiation-Modified Carbon Fibers

The surface activation induced by irradiation has significantly expanded the engineering applications of carbon fibers, particularly in high-performance composite materials. Since the intrinsic mechanical properties of commercial carbon fibers are already close to their theoretical limits, current research primarily aims to enhance interfacial load transfer between carbon fibers and polymer matrices through irradiation-induced surface modification [56,61]. One of the most representative applications is the improvement of interfacial adhesion in carbon fiber-reinforced polymer (CFRP) composites. Poor interfacial bonding often results in interfacial debonding and fiber pull-out, limiting the mechanical performance of CFRPs [62]. Irradiation-induced surface activation provides an effective strategy for overcoming this limitation by simultaneously increasing surface roughness and introducing chemically active functional groups [58]. A representative example was reported by Vautard et al. [63], who developed an EB curing strategy for carbon fiber/acrylate composites. Instead of modifying only the carbon fiber surface, the authors designed a reactive sizing compatible with EB curing, enabling simultaneous irradiation curing and interface strengthening. The resulting composites exhibited an increase in ILSS from 61 MPa to 81 MPa (approximately 33% improvement) without post-curing treatment. Fracture surface analysis demonstrated that failure changed from interfacial debonding to cohesive fracture within the resin matrix, confirming that irradiation effectively strengthened the fiber–matrix interface.

Beyond conventional C-O-C composites, irradiation processing has also been investigated as an alternative manufacturing strategy for high-performance carbon-fiber-reinforced composites, particularly where rapid and low-temperature curing is desirable [64]. Compared with conventional thermal curing, EB irradiation can initiate polymerization and cross-linking within a short processing period while producing relatively little thermal input. For example, Zhao et al. [65] investigated carbon fiber/C-O-C prepregs cured using a 125 keV low-energy electron beam. Even when the irradiation dose reached 300 kGy, the surface temperature of the prepreg increased only to approximately 46.2 °C, demonstrating the intrinsically low-temperature characteristics of EB curing. However, the degree of cure obtained directly after irradiation was only 61.8%, indicating that rapid EB processing does not necessarily guarantee complete matrix cross-linking. Subsequent thermal post-curing increased the degree of cure to 98.5% and the glass-transition temperature to 170.4 °C, demonstrating that a combination of irradiation and optimized post-treatment can provide an effective route for manufacturing CF/C-O-C composites. More recently, Kim et al. [66] systematically investigated the relatively low flexural performance that can occur in EB-cured carbon-fiber composites and proposed a vacuum-bag heat-treatment strategy before electron irradiation. Their results demonstrated that appropriate pre-treatment effectively alleviated the deterioration in flexural properties associated with direct EB curing while retaining the major processing advantage of electron irradiation-namely, reducing the curing process from the several-hour timescale characteristic of conventional thermal processing to the minute timescale. The study therefore highlights an important development in irradiation-assisted CF composite manufacturing: rather than treating irradiation as an isolated replacement for thermal curing, EB processing can be integrated with interface and matrix engineering to balance manufacturing efficiency and mechanical performance.

The curing behavior is also strongly dependent on irradiation parameters. Zhang et al. [67] systematically examined the influence of low-energy EB dose rate on the curing characteristics of carbon fiber/polymer composites, demonstrating that irradiation conditions directly regulate polymerization and therefore affect the final properties of the composite. Such observations emphasize that EB processing should not simply be regarded as a rapid curing method; rather, irradiation dose, dose rate, matrix chemistry, fiber surface state, and subsequent thermal treatment must be jointly optimized to obtain satisfactory composite performance [68]. In addition to irradiation-assisted manufacturing, the radiation resistance of carbon fiber reinforced composite materials is becoming increasingly important in structural applications in high-radiation environments. In this context, irradiation is not regarded as a processing tool but rather an external operating condition that gradually affects the polymer matrix, the fiber-matrix interface, and the macroscopic mechanical response. Hoffman and Skidmore studied carbon fiber reinforced epoxy resin composites exposed to 0.5–2.0 MGy γ-ray doses and found that their modulus, fracture strain, or fracture strength did not show significant changes, although changes caused by irradiation were detected in the epoxy resin matrix [69]. These results indicate that carbon fiber reinforced materials can maintain high mechanical stability while making the polymer matrix more radiation-tolerant.

However, as the cumulative dose increases, the competition between radiation-induced crosslinking and molecular degradation becomes increasingly significant. Liu et al. exposed CF/epoxy resin composites to γ-ray doses of 2, 7, and 20 MGy, and found that their storage modulus, bending strength, and thermal stability began to increase at 2 MGy, but decreased at 7 and 20 MGy [70]. Spectral analysis revealed that both bond breakage and the formation of new oxygen-containing bonds occurred simultaneously in the material, indicating the competitive relationship between crosslinking and degradation reactions. This dose-dependent behavior further proves that radiation resistance cannot be simply described by a monotonic relationship between irradiation dose and it, and supports the existence of an application-related optimal irradiation window.

These considerations are particularly important for the application of materials in aerospace, as carbon fiber reinforced plastics (CFRP) are widely used as lightweight structural materials. However, when exposed to high-energy electrons, protons, ultraviolet radiation, vacuum environments, and thermal cycling conditions for a long time, they may have significant effects [11]. Liu et al. recently investigated CF/epoxy composites subjected to cumulative electron doses of 1000–10,000 kGy and demonstrated strongly orientation-dependent mechanical responses [71]. The tensile strength in the 0° direction increased with the dose, but the corresponding modulus decreased, while the tensile strength in the 90° direction initially increased and then decreased. Scanning electron microscopy (SEM) and X-ray photoelectron spectroscopy (XPS) analyses further revealed the changes in the fiber-matrix interface and chemical bonds caused by irradiation, indicating that long-term electron irradiation not only alters the chemical composition of the matrix but also has a certain impact on the load transfer characteristics at the interface. Fujii and Iwata further evaluated carbon fiber reinforced plastic (CFRP) in a simulated radiation environment of the radio astronomy satellite orbit. They emphasized the significance of the depth-dependent absorbed dose distribution when analyzing the bending performance and the reliability of the spacecraft structure [72]. These studies indicate that the radiation resistance of CFRP used in aerospace not only depends on the stability of the carbon fibers themselves, but is also closely related to matrix degradation, interface evolution, dose distribution, and the orientation of the reinforcing materials.

Similar issues also arise in nuclear environments, where carbon fiber reinforced polymers (CFRP) may experience significant cumulative gamma-ray doses, and depending on the specific application, they may also be affected by mixed radiation fields. High-dose irradiation studies have shown that the polymer matrix and the fiber-matrix interface are often the most radiation-sensitive regions in composite materials. This behavior has also been observed in three-dimensional woven CFR/epoxy resin composites, where gamma-ray-induced damage is distributed in the matrix, interface, and near-interface regions, while the degree of mechanical performance degradation strongly depends on the weaving structure [15]. Importantly, the interface was proposed to act as a region capable of capturing irradiation-induced defects, suggesting that interface design may influence not only mechanical load transfer but also the spatial development of radiation damage.

More recently, radiation-resistant composite design has shifted from passive damage evaluation toward active matrix and interface engineering. Yan et al. introduced carbon nanotubes into the epoxy matrix and fiber–matrix interfacial region and demonstrated improved γ-radiation resistance of CF/epoxy composites [73]. When CNTs were simultaneously incorporated into both regions, the irradiated composites exhibited substantially higher bending strength, bending modulus, and storage modulus than the unmodified system, together with reduced internal damage after fatigue testing. These results demonstrate that radiation resistance can be enhanced through deliberate modification of both the matrix and interface rather than relying solely on the intrinsic stability of the carbon fibers.

Therefore, the irradiation-related engineering of carbon fiber composite materials can be divided into two complementary paths. The first one is irradiation-assisted processing, which intentionally uses electron beams or γ-rays to activate the surface, enhance the interface, and achieve rapid curing of the matrix. The second one is anti-irradiation structural design, which involves engineering design to ensure that carbon fiber reinforced plastics can maintain their mechanical properties and interface characteristics when exposed to high radiation environments for a long time in aerospace, nuclear energy, and other fields. Combining these two paths can provide a more complete framework for understanding the role of irradiation in carbon fiber composite materials and highlights the necessity of establishing quantitative relationships between irradiation conditions, matrix chemical composition, interface evolution, composite material structure, and residual mechanical properties.

### 2.4. Activated Carbon and Biochar

#### 2.4.1. Radiation-Induced Structural Evolution

AC and biochar are porous carbonaceous materials generally produced through the thermochemical conversion of carbon-rich precursors. Although both materials contain disordered aromatic carbon domains, their structures and physicochemical properties strongly depend on precursor composition and preparation conditions. AC is typically subjected to additional physical or chemical activation, resulting in a highly developed micro-/mesoporous structure and large specific surface area, whereas biochar generally retains a more heterogeneous carbon framework containing aromatic domains, oxygen-containing functional groups, residual minerals, and precursor-derived heteroatoms. These characteristics distinguish their irradiation responses from those of graphene and carbon nanotubes: instead of producing isolated atomic defects within a well-defined sp^2^ lattice, irradiation primarily regulates the pore architecture, degree of structural disorder, surface functional groups, and interfacial chemistry of AC and biochar [74,75].

**Figure 7:**
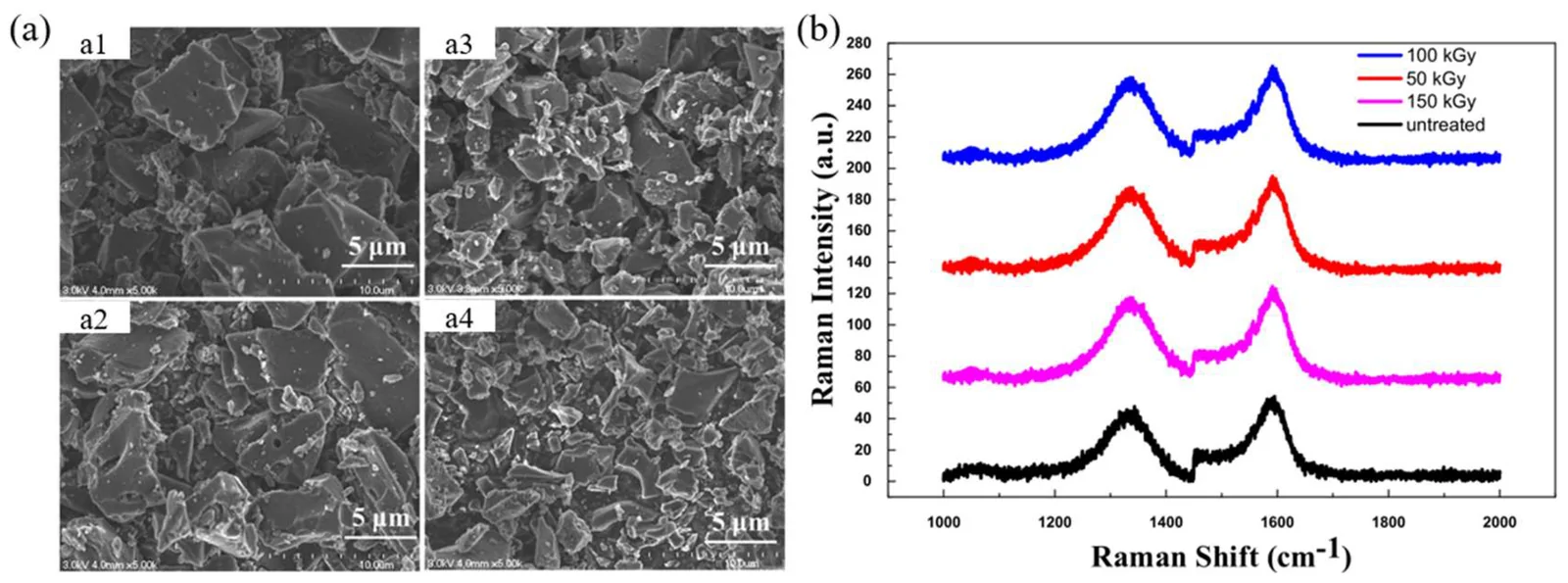
Structural and morphological evolution of biochar under γ-ray irradiation. (**a1**) untreated biochar, (**a2**) 50 kGy gamma activated, (**a3**) 100 kGy gamma activated, and (**a4**) 150 kGy gamma activated biochar. (**b**) Raman spectra of untreated biochar and biochar irradiated at 50, 100, and 150 kGy. (Adapted from Adhamash et al. [75]).

Exposure to γ rays or high-energy electrons can induce bond cleavage and structural rearrangement within the disordered carbon matrix, thereby altering particle size, pore accessibility, and the relative proportion of ordered and defective carbon domains. Importantly, the irradiation response is strongly dose-dependent. Moderate irradiation can open previously inaccessible pores or generate additional defects, whereas excessive irradiation may destroy pore walls or induce structural rearrangement, leading to deterioration of the desired properties [16]. Adhamash et al. [75], for example, irradiated biochar carbon YP-50 at 50, 100, and 150 kGy and observed pronounced dose-dependent morphological changes. SEM images (Figure 7a) showed progressive fragmentation and restructuring of the carbon particles, while BET measurements revealed that the specific surface area increased from 1451 m^2^ g^−1^ for untreated carbon to 1563 m^2^ g^−1^ at 100 kGy, accompanied by an increase in micropore volume from 0.46 to 0.55 cm^3^ g^−1^. When the dose was further increased to 150 kGy, however, the surface area decreased to 1511 m^2^ g^−1^, demonstrating the existence of an optimum irradiation window rather than a simple linear dose structure relationship. Raman spectroscopy (Figure 7b) provides complementary evidence for irradiation-induced structural disorder. In the same study, the characteristic D and G bands remained visible after γ irradiation, indicating preservation of the overall carbonaceous framework; however, the *I_D_/I_G_* ratio increased from 0.833 for untreated carbon to 0.843 at 100 kGy, consistent with the generation of additional defective sites. These results indicate that appropriately controlled γ irradiation can simultaneously increase structural disorder and improve pore accessibility without completely disrupting the conductive carbon network.

Surface chemistry represents another important component of irradiation-induced evolution. When irradiation is conducted in the presence of water, oxygen, ammonia, or other reactive species, radiolysis generates highly reactive radicals that can interact with the carbon surface, enabling oxidation, reduction, heteroatom incorporation, or graft polymerization. For example, γ-radiolysis has been used to introduce nitrogen functionalities into biomass-derived AC through ammonia treatment without the high-temperature processing normally required for conventional N-doping. Such modification changes surface polarity and adsorption-site chemistry and therefore provides a direct bridge between irradiation-induced structural evolution and subsequent functional applications [74].

#### 2.4.2. Applications of Irradiation-Modified AC and Biochar

The irradiation-induced regulation of pore architecture and surface chemistry has enabled AC and biochar to exhibit enhanced performance in both environmental remediation and electrochemical energy storage. Unlike graphene, where environmental applications are predominantly associated with surface functionalization, porous carbons benefit simultaneously from changes in pore accessibility, surface area, defect density, and chemical functionality. Consequently, irradiation can substantially alter both physical adsorption and surface-specific interactions with target species [76].

**Figure 8:**
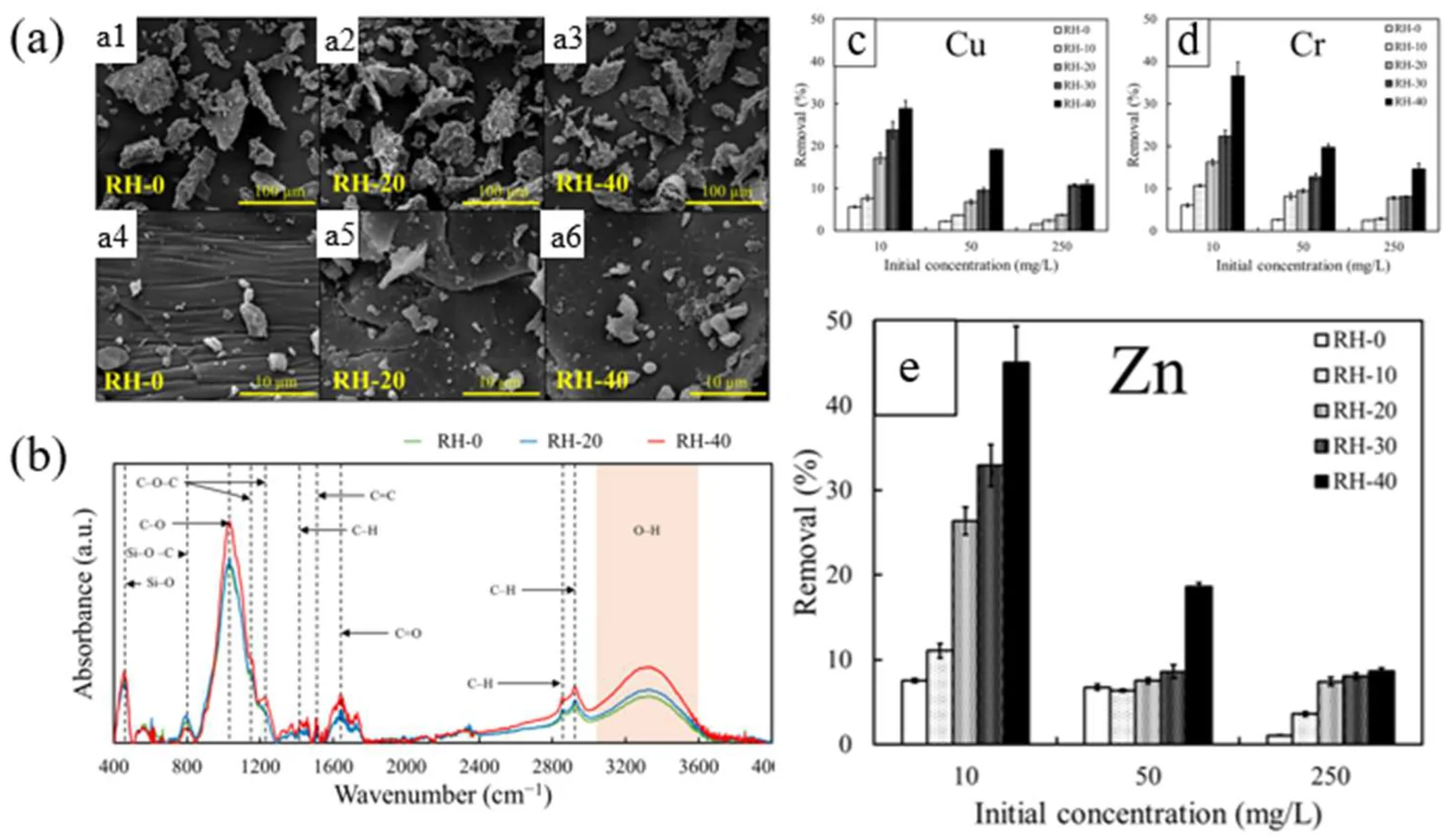
γ-Irradiation-induced surface modification and enhanced heavy-metal adsorption of a biomass-derived rice-husk biosorbent. (**a**) Representative SEM images of non-irradiated and γ-irradiated rice husk. (**b**) FTIR spectra showing irradiation-induced changes in surface functional groups. (**c**–**e**) Removal efficiencies of Cu^2+^, Cr^3+^, and Zn^2+^by rice husk subjected to different γ-irradiation doses. (Adapted from Saemood et al. [77]).

Recently, Saemood et al. [77] reported a representative environmental application study. They investigated γ-ray irradiation as a surface modification strategy without the need for reagents, for the removal of heavy metals from rice husk-derived bioadsorbents. The samples were exposed to ^60^Co γ-rays at doses ranging from 0 to 40 kGy, and their structure, chemical properties, and adsorption performance were systematically evaluated. Interestingly, scanning electron microscopy (SEM) and BET analysis revealed that the morphology and pore characteristics of the samples only underwent relatively minor changes after irradiation, while Fourier transform infrared spectroscopy (FTIR) and elemental analysis results indicated that with the increase in irradiation dose, oxygen-containing functional groups gradually strengthened on the surface of the samples (Figure 8a,b). These chemical modifications significantly enhanced the adsorption capacity for Cu^2+^, Cr^3+^, and Zn^2+^, and at a dose of 40 kGy, the removal performance of the samples for these three metal ions reached the highest level (Figure 8c–e). At an initial concentration of 10 mg^−1^, compared to the unirradiated materials, the 40 kGy sample’s removal efficiency for Cu^2+^, Cr^3+^, and Zn^2+^ increased by approximately 415%, 502%, and 663%, respectively. These results indicate that the enhanced adsorption effect due to irradiation in biomass-derived carbon-based adsorbents does not necessarily require extensive pore restructuring; instead, controlled surface oxidation and the introduction of oxygen-containing functional groups play a dominant role in improving the binding of metal ions.

More direct evidence for irradiation-assisted surface functionalization has been obtained using AC. Biomass-derived ACs prepared from water hyacinth and eucalyptus charcoal were treated with ammonia under γ irradiation to produce nitrogen-modified adsorbents [74]. In contrast to conventional hydrothermal nitrogen modification, γ radiolysis enabled nitrogen incorporation without an additional high-temperature treatment. The resulting materials were subsequently evaluated for methylene blue removal, demonstrating that radiation chemistry can be used not only to create structural defects but also to introduce targeted surface functionalities for contaminant adsorption. This is particularly important for porous carbon materials because adsorption performance is governed by both available surface area and the chemical affinity of individual adsorption sites [78].

More recently, this strategy has been extended toward multi-metal wastewater remediation. A 2026 study investigated sawdust-derived AC subjected to γ doses between 0 and 40 kGy for the removal of Cu^2+^, Cr^3+^, and Zn^2+^ [79]. Gamma irradiation introduced additional oxygen-containing surface functionalities, and the materials irradiated at 40 kGy exhibited the highest adsorption capacities for all three metals. Adsorption analysis indicated that chemisorption played a major role, directly connecting irradiation-induced surface chemistry with enhanced heavy-metal capture. This example is especially useful for the present review because it demonstrates that radiation treatment can tune a conventional low-cost AC toward simultaneous multi-metal remediation, rather than merely improving adsorption of a single model contaminant.

The functional benefits of irradiation are not limited to environmental adsorption. Electrochemical energy storage represents another particularly convincing application because the irradiation-induced changes in pore architecture can be directly translated into ion transport and charge-storage performance. In addition to environmental remediation, porous activated carbon and biochar have also become highly attractive electrode materials in the field of electrochemical energy storage due to their high specific surface area, interconnected pore networks, and controllable surface chemical properties. The strong dependence of electrochemical performance on pore architecture and surface chemistry has been widely established for biomass-derived porous carbons [80,81], providing the physicochemical basis for understanding why irradiation-induced regulation of these features can improve charge-storage behavior. Adhamash et al. [75] used γ-irradiated biochar carbon as an electrode for electric double-layer capacitors and observed a pronounced optimum at 100 kGy. The specific capacitance increased from 115.3 F g^−1^ for untreated biochar to 246.2 F g^−1^ after irradiation, corresponding to more than a two-fold enhancement. At the same time, charge-transfer resistance decreased from 21.7 to 7.4 Ω, indicating substantially improved charge transport. The optimized electrode delivered an energy density of 34.2 Wh kg^−1^ and retained more than 96% of its capacitance after 10,000 cycles. The improvement was associated with the increased surface area, enlarged pore volume, smaller particle dimensions, and improved conductivity generated at the optimized irradiation dose. This example also highlights an important feature of irradiation modification: higher radiation doses do not necessarily produce better performance. Although irradiation at 100 kGy generated an optimized porous structure, increasing the dose to 150 kGy reduced the surface area and electrochemical performance. A recent study further demonstrated this concept using rice-husk-derived AC subjected to γ irradiation for supercapacitor applications [16]. The work showed that radiation treatment modified the carbon structure and electrochemical response, providing additional evidence that irradiation-assisted pore and defect engineering is transferable across different biomass-derived porous carbons rather than being restricted to a single commercial biochar system.

Overall, irradiation directly regulates defect engineering in AC and biochar through the coupled modification of carbon disorder, pore structure, and surface chemical states. Irradiation-induced bond cleavage and radical reactions can generate new defect sites and alter the population of oxygen- or nitrogen-containing functional groups, while structural rearrangement can modify pore accessibility and the available surface area. At an appropriate irradiation dose, these changes increase the number and accessibility of active sites, thereby facilitating adsorbate–surface interactions in environmental remediation and ion adsorption/transport in electrochemical energy storage. However, excessive irradiation may cause defect accumulation, pore deterioration, or partial structural degradation, offsetting these benefits. Therefore, the functional response of porous carbons is governed by an irradiation-dependent balance between beneficial defect generation and excessive structural damage, further supporting the existence of an optimal irradiation window for defect-engineered carbon materials.

Finally, in order to facilitate a direct comparison of different carbon structures, we have attached Table 2, which summarizes the representative irradiation studies discussed previously. The table covers the irradiation sources and doses, the main structural and performance changes caused by irradiation, as well as the underlying mechanisms. This further highlights the dependence of irradiation engineering on the structure.

## 3. Conclusions and Future Work

Irradiation provides a versatile approach for tailoring the structure and properties of carbon-based materials without fundamentally changing their original material systems. This review summarizes the irradiation responses and representative applications of four typical carbon materials, including graphene-based materials, carbon nanotubes, carbon fibers, and AC/biochar. Although these materials are all predominantly composed of carbon, their responses to irradiation differ considerably because of their distinct structural characteristics. In graphene, irradiation primarily generates vacancies, reconstructed defects, and surface functionalization, which can subsequently regulate adsorption and interfacial interactions. Carbon nanotubes exhibit not only conventional point-defect formation but also characteristic inter-tube cross-linking and welding, providing an effective route for strengthening carbon nanotube assemblies and tailoring their electronic properties. For carbon fibers, irradiation mainly affects surface chemistry and the fiber–matrix interface rather than the bulk carbon structure, making interface engineering an important pathway for improving composite performance. In contrast, AC and biochar exhibit pronounced changes in pore accessibility, structural disorder, and surface functional groups, which directly influence their adsorption and electrochemical properties. Therefore, the effects of irradiation on carbon materials cannot be simply generalized as radiation damage; depending on the material structure and irradiation conditions, controlled radiation exposure can be intentionally utilized as a defect-, surface-, pore-, or interface-engineering strategy.

Despite these promising results, several issues still restrict the controllable application of irradiation modification. Most importantly, the relationship between irradiation conditions, generated structures, and resulting properties remains insufficiently understood. Irradiation dose is frequently used as the primary control parameter, whereas the effects of radiation type, energy, dose rate, atmosphere, temperature, and initial carbon structure are not always systematically considered. Moreover, the structural characterization of irradiated carbon materials still relies heavily on Raman spectroscopy and XPS. Although these techniques can indicate changes in disorder and surface chemistry, they cannot independently identify the exact nature and spatial distribution of irradiation-induced defects. Consequently, improved performance is often broadly attributed to increased defect density even when several structural changes-including vacancies, functional groups, pore evolution, and interfacial reconstruction-occur simultaneously. Another common feature identified throughout the studies discussed above is the competition between beneficial modification and excessive radiation damage. Increasing irradiation dose does not necessarily continuously improve performance; beyond an appropriate irradiation window, excessive disorder, framework degradation, or deterioration of electrical and mechanical properties may occur. Similar dose-dependent defect evolution and structural reconstruction have been widely reported for irradiated carbon systems.

Future studies should therefore focus less on simply demonstrating that irradiation can improve material performance and more on establishing quantitative irradiation–structure–property relationships. A particularly important direction is to determine the type and concentration of defects required for a specific function. For graphene, the relative contributions of vacancies and irradiation-induced surface functional groups should be distinguished; for carbon nanotubes, the optimal balance between inter-tube cross-linking and nanotube-wall damage needs to be identified; for carbon fibers, surface activation should be maximized without compromising the intrinsic strength of the fiber; and for AC and biochar, changes in pore accessibility should be separated from changes in surface chemistry. Combining Raman and XPS with atomic-resolution TEM/STEM, together with BET analysis for porous carbons, would provide more direct evidence for these relationships. In parallel, systematic comparisons using the same starting carbon material under γ-ray, EB, and ion irradiation would help clarify how different energy-deposition mechanisms control defect formation, surface reactions, and structural reconstruction. The distinct responses produced by different irradiation sources have already been recognized in carbon and 2D materials, but direct experimental comparisons under equivalent conditions remain limited. Ultimately, the key objective should be to transform irradiation from an empirical post-treatment method into a controllable carbon-material engineering strategy. Rather than pursuing the highest defect density or irradiation dose, future irradiation design should target the specific structural feature required by the intended application.

## Figures and Tables

**Table 1:** Comparative characteristics and typical structural effects of major irradiation sources in carbon-based materials.

Irradiation Source	Dominant Interaction	Typical Defects/Modifications	Key Characteristics	Main Effects/Applications
γ-rays	Ionization and electronic excitation	Functional groups, radicals, structural disorder	High penetration; relatively uniform treatment	Surface functionalization, grafting, pore modification
Electron beam	Electronic excitation and knock-on displacement	Vacancies, divacancies, reconstructed defects	Energy-dependent and controllable	Defect engineering, CNT cross-linking, composite curing
Ion beam	Nuclear collisions and electronic energy loss	Vacancies, interstitials, defect clusters	Localized damage; depth-dependent	Defect engineering, implantation, local structural modification

**Table 2:** Representative irradiation conditions, structural modifications, performance changes, and dominant mechanisms in carbon-based materials.

Carbon Material	Irradiation Source	Irradiation Dose	Chemical Modification	Performance Improvement	Dominant Mechanism
Graphene/ GO [27]	^60^Co γ-rays	Dose-dependent γ-ray	Vacancies, point defects, reconstructed defects; increased oxygen functionalities	Transition from crystalline graphene toward defective/nanocrystalline structure	Atomic displacement + defect-assisted oxygen adsorption
Graphene/GO [35]	Electron beam	5–40 kGy	Regulation of oxygen-containing groups and C/O ratio	Maximum Pb(II) adsorption capacity: 194.76 mg g^−1^ at 5 kGy	Irradiation-controlled surface chemistry enhances Pb(II) binding
GO [37]	γ-rays	Irradiation-assisted radical polymerization	Surface grafting/functionalization and increased defect-related disorder	Enhanced chemical tunability and adsorption functionality	Radiation-generated radicals initiate surface grafting
CNT yarn [49]	Electron beam	EB irradiation with AA/AN	Defects, functionalization and inter-tube cross-linking; reduced voids	Tensile strength: 251.1 → 444.5 MPa; modulus: 8.79 → 21.5 GPa	Covalent cross-links suppress inter-tube sliding and improve load transfer
CNT FET [51]	γ-rays	Up to 155 kGy	Limited damage in CNT channel; radiation response dominated by substrate/interface	Stable transistor characteristics up to ≥155 kGy	Intrinsic radiation tolerance of sp^2^ CNT network and partial substrate shielding
CNT CMOS [50]	γ-rays	60 kGy(Si)	CNT network largely preserves electronic transport	Logic gates and ring oscillators retain normal operation with minimal delay variation	Radiation-hard CNT channels and optimized device architecture
Carbon fiber/epoxy [59]	^60^Co γ-rays	Dose-dependent	Surface defects, roughening and increased oxygen-containing groups	Moderate irradiation improves ILSS; excessive irradiation causes degradation	Surface activation improves wettability and fiber–matrix bonding
CF/acrylate composite [63]	Electron beam	EB curing	Reactive interface formation/cross-linking	ILSS: 61 → 81 MPa (~33%)	Irradiation curing + engineered interface chemistry enhance load transfer
CF/epoxy prepreg [65]	125 keV electron beam	Up to 300 kGy	Matrix polymerization/cross-linking	Degree of cure: 61.8% after EB; 98.5% after post-curing	Radiation-induced polymerization followed by thermal completion
CF/epoxy [70]	γ-rays	2, 7, 20 MGy	Competing cross-linking, bond scission and oxidation	Mechanical/thermal properties improve at 2 MGy but decline at 7–20 MGy	Competition between radiation-induced cross-linking and degradation
Biochar carbon YP-50 [75]	γ-rays	50–150 kGy; optimum 100 kGy	Increased disorder, pore accessibility and micropore volume	SSA: 1451.2 → 1562.9 m^2^ g^−1^; capacitance: 115.3 → 246.2 F g^−1^	Optimized pore/defect structure improves ion accessibility and charge transport
Rice-husk biosorbent [77]	^60^Co γ-rays	0–40 kGy	Increased oxygen-containing surface functionalities; minor pore change	Cu^2+^, Cr^3+^ and Zn^2+^ removal increased by approx. 415%, 502%, 663% at 40 kGy	Surface oxidation increases metal-binding sites
Biomass-derived AC [74]	γ-rays	γ irradiation in ammonia	N-containing surface functionalities	Improved methylene-blue adsorption functionality	Radiolysis-driven N incorporation modifies adsorption-site chemistry
Sawdust-derived AC [79]	γ-rays	0–40 kGy	Increased oxygen-containing surface groups	Highest Cu^2+^, Cr^3+^ and Zn^2+^ adsorption at 40 kGy	Surface functionalization promotes chemisorption

## Data Availability

No new data were created or analyzed in this study. Data sharing is not applicable.
